# Effects of reducing the number of candidate tasks in voluntary task switching

**DOI:** 10.3389/fpsyg.2014.01555

**Published:** 2015-01-06

**Authors:** Thomas Kleinsorge, Juliane Scheil

**Affiliations:** Leibniz Research Centre for Working Environment and Human FactorsDortmund, Germany

**Keywords:** cognitive control, task switching, Cueing, executive functions, preparation

## Abstract

Recently, Demanet and Liefooghe (2014; Experiment 3) reported an experiment on voluntary task switching (VTS) in which the number of candidate tasks to choose from was reduced from 4 to 2 before participants indicated their task choice. This procedure was intended to prevent participants from choosing a task in advance of the presentation of a prompt to do so. This procedure is highly similar to a procedure recently employed by Kleinsorge and Scheil (2013) in a study of cued task switching which yielded evidence for a selective facilitation of task switches by a reduction of the number of tasks to two. In order to examine whether a similar effect would also be observed with VTS, we conceptually replicated the experiment of Demanet and Liefooghe (2014) with an additional control condition in which the number of tasks was not reduced. In this experiment, no evidence for a facilitation of task switching could be observed, pointing to a functional divergence between explicit task cues and the internally generated cues involved in VTS. In addition, we observed evidence for a selective advantage of forced switch trials over repetition-possible trials that was largely independent of the duration of the preparation interval. This effect was accompanied by a massive increase of task indication times in conditions with a reduced number of tasks, suggesting that this manipulation resulted in a pronounced change in the way participants performed voluntary task switches.

## INTRODUCTION

Research on task switching is intimately linked to controversies regarding suitable measures of cognitive control processes (cf. Kiesel et al., 2010; Vandierendonck et al., 2010, for reviews). A large part of the corresponding empirical efforts centers around the disentanglement of top–down and bottom–up factors as being reflected in switch costs, that is, increases of reaction times (RTs) and error rates (ERs) observed in task switch trials as compared to task repetition trials. Whereas top–down factors like proactive preparation are related to cognitive control proper, bottom–up factors like interference induced by a previously performed task are more related to a need for control.

The majority of task switching experiments employs some version of cued task switching. With these procedures, the task to-be performed in each trial is indicated by an external cue that guides the performance of participants. Unfortunately, many observations obtained with this procedure as indications of top–down control have been shown to be open to alternative explanations in terms of stimulus-based, bottom–up factors (e.g., Schneider and Logan, 2005). This has led Arrington and Logan (2004, 2005) to introduce voluntary task switching (VTS) as a procedure to increase the potential influence of top–down processes. In VTS, participants are free (within certain constraints related, for example, to the proportion of task switches) to choose from a number of candidate tasks in each trial. Because this procedure gets along without explicit task cues, it is easy to rule out cue-related processes that have been established as alternatives to top–down processes as an explanation for (some proportion of) switch costs.

However, even with VTS a number of bottom–up factors have been shown to affect task switching performance. For instance, stimuli associated with a certain task in a previous trial have been shown to be more likely to induce a choice of the same task than an alternative task in a subsequent trial (Arrington et al., 2010). Therefore, one needs to take into account bottom–up factors also with VTS.

Recently, Demanet and Liefooghe (2014) reported a series of experiments aimed at separating top–down and bottom–up processes involved in VTS by simultaneously varying the interval between task execution in trial *n* – 1 and the onset of the cue prompting the task-indication response in trial *n* on the one hand, and the interval between the task-indication response and the onset of the imperative stimulus on the other hand. The first interval, which the authors termed ‘execution response – prompt interval’ (ERPI), was intended to manipulate bottom–up factors, whereas the second interval (‘indication response – stimulus interval’, IRSI) was intended to vary the extent of top–down preparation. Specifically, comparing conditions with both a short ERPI and IRSI with conditions with a long ERPI and a short IRSI should yield a measure of the dissipating influence of bottom–up processes, whereas comparing conditions with a long ERPI and a short IRSI with conditions with a short ERPI and a long IRSI should provide a measure of the impact of top–down processes. (In the latter case, the sum of both intervals was constant, resulting in equal amounts of passive dissipation across the two conditions.)

Whereas the first experiment of Demanet and Liefooghe (2014) seemed to provide evidence for both a bottom–up and a top–down component, the observations from two additional experiments strongly suggested that the evidence for a top–down component observed in the first experiment was due to participants initiating task preparation in advance of the overt task indication response. For the present purposes, Experiment 3 is the most important one. In this experiment, there were four candidate tasks. This set of four tasks was reduced to two by the task indication prompt. Because participants did not know which two tasks were actually eligible in a certain trial, this procedure strongly discouraged premature preparation for one of the tasks. Under these conditions, the reduction of RT by a long IRSI was equally pronounced with task repetitions and task switches, that is, there was no reduction of switch costs when the IRSI was prolonged from 100 to 2,000 ms. Because a reduction of switch costs as a function of a longer preparation interval (known as the RISC effect) is considered as a hallmark of top-down processes in task switching (cf. Monsell and Mizon, 2006), Demanet and Liefooghe (2014) concluded not to have observed any indication of top–down processes in VTS.

However, the procedure employed by Demanet and Liefooghe (2014) in their Experiment 3 might have induced some top–down processes that went unnoticed due to a lack of the proper control condition. This consideration is based on a recent study from our lab (Kleinsorge and Scheil, 2013) in which we also employed a manipulation by which a set of four candidate tasks was reduced to only two on a trial-to-trial basis, but this manipulation only affected half of the trials whereas in the remaining trials, all four tasks remained candidate tasks until a task cue ultimately determined the relevant task for the present trial. Thus, in contrast to the VTS procedure of Demanet and Liefooghe (2014), this study employed a modified version of cued task switching. The main observation of these experiments consisted of a reduction of RTs induced by a reduction of the set of candidate tasks from 4 to 2 that predominantly affected switch trials. We proposed that this was due to the establishment of antagonistic constraints among the remaining two candidate tasks that would enable task selection to be based on a rather superficial processing of the task cue, compared to conditions in which selection was among four tasks that do not allow for an establishment of antagonistic constraints among the candidate tasks. Antagonistic constraints exploit the fact that evidence for one of two tasks directly translates into evidence against the other one and vice versa, which is not the case when more than two tasks are possible candidate tasks. Antagonistic constraints are, for example, implemented in diffusion models of decision processes which assume that the decision process is characterized by a continuous sampling of information in a way that every piece of information that drives the decision process toward a decision in favor of one of the alternatives simultaneously drives it away from the alternative decision (cf. Schmitz and Voss, 2014, for an application to task switching). Such reciprocity is not given with decisions between more than two alternatives because evidence against one of the alternatives does not directly translate into evidence in favor of one of the other alternatives.

Based on the structural similarities between the experiments of Kleinsorge and Scheil (2013) and Experiment 3 of Demanet and Liefooghe (2014), this line of reasoning may suggest that participants of Demanet and Liefooghe’s (2014) Experiment 3 may have engaged in top–down processing in terms of an establishment of antagonistic constraints, which may have gone unnoticed due to the lack of a control condition in which there were four candidate tasks to choose from. Of course, this presupposes that an establishment of antagonistic constraints also plays a role in VTS. As will be outlined afterward, there are reasons to doubt this assumption, so the present study was primarily designed to explore whether what was observed by Kleinsorge and Scheil (2013) with cued task switching would also be observed with VTS.

The notion of antagonistic constraints rests on the assumption that switching among (only) two tasks allows for a kind of shortcut in the process of task selection that exploits the fact that a selection among two tasks can be based on any single feature that distinguishes between the two tasks. In terms of information theory, only one bit of information has to be processed in order to distinguish between two tasks. Originally, the notion of antagonistic constraints was introduced by Kleinsorge and Apitzsch (2012) to account for the observation that precue-based task switching is associated with superior performance compared to memory-based task switching with two but not with four tasks. The basic idea was that the combination of switching among two tasks and precue-based switching results in some kind of compatibility between processing and coding requirements in this situation. This compatibility arises because the distinction between tasks in terms of physical features inherent in precue-based task switching meshes well with the antagonistic constraints inherent in choices between two tasks. In contrast, switching on the basis of memory for a sequence of tasks should be aided by an elaborated representation of the tasks that codes many interrelations among them, being more compatible with switching among a larger set of tasks that does not allow for an establishment of a single antagonistic constraint in principle.

According to this reasoning regarding the link between antagonistic constraints and cued task switching among two tasks, the question whether antagonistic constraints play a role in VTS leads to the question of functional equivalence of external task cues and internally generated task cues in VTS. For the present purposes, functional equivalence would yield evidence indicative of an establishment of antagonistic constraints also with VTS. In contrast, a lack of such evidence would hint at a functional divergence between externally presented and internally generated task cues.

To examine this question, we conceptually replicated Experiment 3 of Demanet and Liefooghe (2014) but added an additional control condition in which participants could choose among the whole set of four tasks. Observing a selective increase of RTs in switch trials with four candidate tasks would point to an establishment of antagonistic constraints. This, in turn, would argue for a functional equivalence of external task cues on the one hand and internally generated selection cues in VTS on the other hand. Such a selective increase of RTs in switch trials should probably occur across all combinations of ERPIs and IRSIs. The latter assumption is based on the consideration that antagonistic constraints are probably established concurrently with the reduction of the set of candidate tasks from 4 to 2. Furthermore, in our original study (Kleinsorge and Scheil, 2013, Experiment 1) the selective decrease of RTs for task switches by a reduction of the number of tasks was not affected by the length of the task precuing interval.

In addition to adding a control condition in which the number of candidate tasks remained four, we employed a between-participants variation of the proportion of trials in which a task repetition was among the candidate tasks when the set of tasks was reduced to two. This proportion was 0.25 in the experiment of Demanet and Liefooghe (2014), but 0.5 in Experiment 1 of Kleinsorge and Scheil (2013). Although our previous study also included a control experiment with a proportion of 0.25 of task-repetition possible trials with a reduced set of tasks, this experiment yielded a somewhat ambiguous interaction of reducing the number of tasks with the duration of the precuing interval. Therefore, we decided to include this factor in the present study to rule out any ambiguities based on changes in task expectancy that may accompany the reduction of the number of tasks. (cf. Kleinsorge and Scheil, 2013, for a detailed discussion of this point. To anticipate results, this variable was of no particular importance with respect to the present study. Therefore, it will not be considered in more detail at this point.)

To summarize our main hypotheses, we first expected to replicate the basic findings of Demanet and Liefooghe (2014) in terms of evidence for a substantial bottom–up component but no top–down mediated reduction of switch costs in a VTS procedure that strongly discouraged participants from task preparation in advance of a task indication response. Because we used a different set of tasks and implemented a control condition that allowed for task preparation in advance of the task indication response, a successful replication would considerably strengthen the empirical basis with respect to this issue. Our main interest, however, related to the interaction of restricting the number of tasks and task switching. Observing a reduction of switch costs by restricting the number of tasks from 4 to 2 would provide evidence for an establishment of antagonistic constraints also in VTS. Note that this hypothesis relates to a first-order interaction of task choice and task transition that is not confined to any subset of conditions, for example, does not depend on a specific combination of ERPI/IRSI.

## MATERIALS AND METHODS

### PARTICIPANTS

Eighty right-handed subjects participated. One subject had to be excluded due to missing observations in some conditions because of always choosing task switches in the restricted condition. The final sample consisted of 17 men and 62 women with a mean age of 23.7 years (range: 19–30). All had normal or corrected-to-normal vision. They were assigned to one of the two groups according to their order of appearance according to an odd–even scheme.

### STIMULI, TASKS, AND APPARATUS

Imperative stimuli consisted of the digits 2 and 7 written in either blue or yellow and in either Arial or Batang font. Each digit was about 7 mm high × 4 mm wide. There were four tasks in total: digits had to be judged regarding their magnitude (smaller vs. larger than five), parity, color, or font. Task indication responses were made by pressing one of the keys “a” (magnitude), “s” (parity), “d” (font), or “f” (color) of a German QWERTZ keyboard with the left hand. Task execution responses were made with the right hand by pressing the “j”-key for small, uneven, blue, and Arial stimuli and the “k”-key for large, even, yellow, and Batang stimuli.

Stimuli were presented centrally on a 17′′ monitor on black background. Viewing distance was not controlled, but amounted to ∼60 cm.

### PROCEDURE

The procedures employed in the present study were approved by the ethics committee of the Leibniz Research Centre for Working Environment and Human Factors. After giving informed consent, participants were provided with on-screen instructions. They were informed that they had to select one task at the beginning of each trial and that in half of the blocks, they could freely choose between all four tasks whereas in the other half, only two tasks were allowed to choose from in each trial. Participants were instructed to select all tasks equally often in random order. A strictly random order would correspond to a switch proportion of 75% of the trials, which conforms to the procedure of Demanet and Liefooghe (2014; Experiment 3). Furthermore, because we noticed during pilot testing that some participants tended to switch the task in a much larger proportion of trials, we employed a feedback procedure (see below) to induce participants to conform to a switch proportion within a range of 65–85% of trials.

The experiment consisted of 16 blocks of 96 trials each. Blocks in which participants chose among all four possible tasks and blocks in which task choice was restricted to two tasks per trial alternated. Half of the participants started with a restricted block, the other half with an unrestricted one. At the end of each block, participants received feedback (in percentage terms) about how often each task was chosen and how many task switches they made. If the proportion of task switches was below 65% of trials, participants were instructed on screen to switch tasks more often. If the proportion of task switches was above 85%, they were instructed to repeat tasks more often.

There were two groups that differed regarding the percentage of possible task repetitions in the restricted condition. For one group, the restricted set of tasks contained a task repetition in 25% of the trials, whereas for the second group, 50% of trials allowed for a task repetition.

Three different timing conditions were employed (following Demanet and Liefooghe, 2014): ERPI = 100 ms/IRSI = 100 ms, ERPI = 2,000 ms/IRSI = 100 ms, and ERPI = 100 ms/IRSI = 2,000 ms. A comparison of condition ERPI = 100/IRSI = 100 with condition ERPI = 2,000/IRSI = 100 should yield a measure of the dissipation of bottom–up influences, whereas a comparison of condition ERPI = 2,000/IRSI = 100 with condition ERPI = 100/IRSI = 2,000 should yield a measure of top–down control (cf. Demanet and Liefooghe, 2014, for a more detailed account).

**FIGURE 1:**
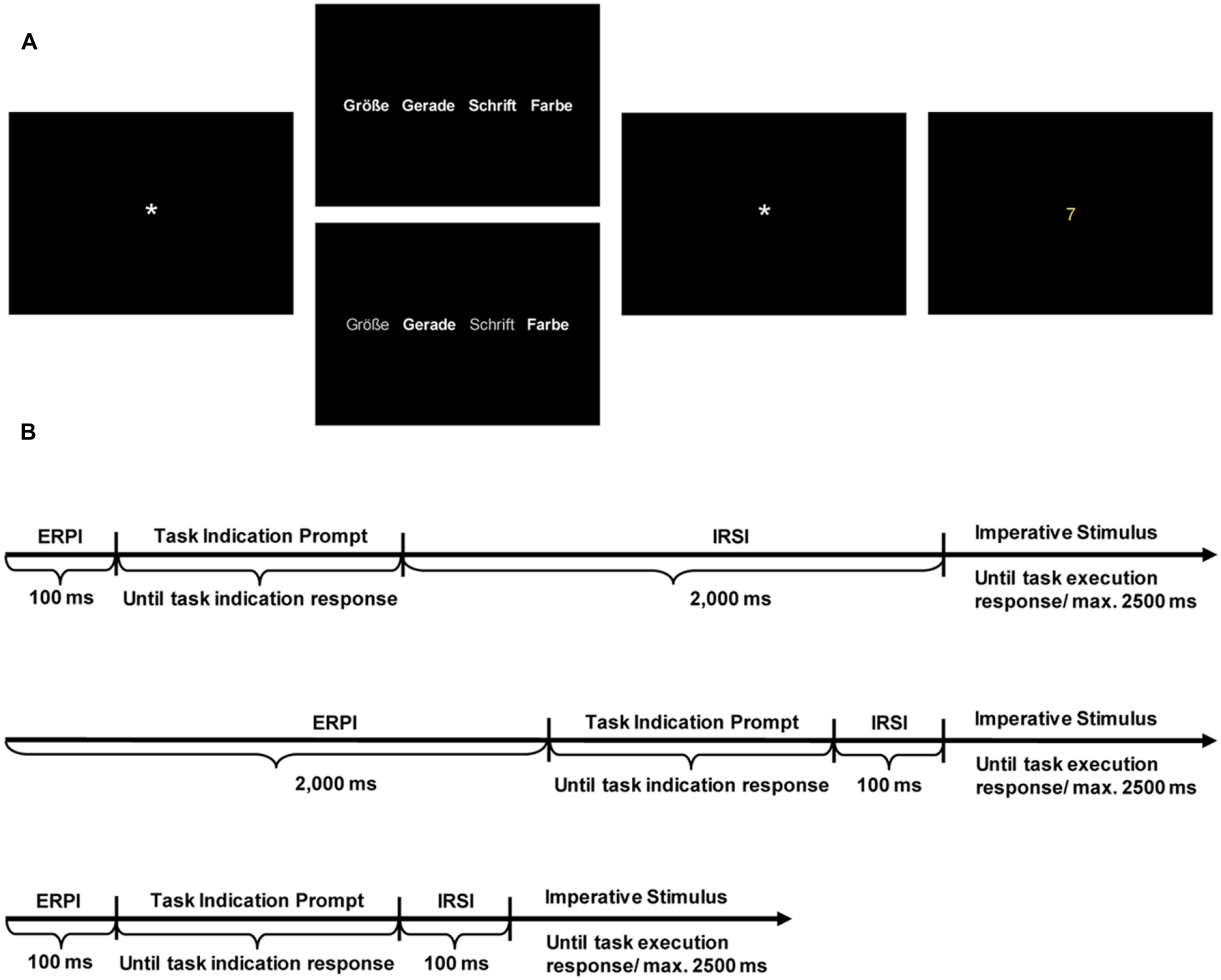
**(A)** Schematic illustration of the displays used in a single trial (in chronological order). Asterisk indicates fixation mark. **(B)** Schematic illustration of the procedure of single trials.

Each trial began with a fixation mark presented for the length of the ERPI. After that, the German names of the four tasks (“Größe” for magnitude, “Gerade” for parity, “Schrift” for font, “Farbe” for color) were visible on the screen until participants made a task indication response by pressing one of four keys (a, s, d, or f, see above) with their left hand. If all four tasks could be chosen, all tasks names were presented in white. In case of a task restriction, the two candidate tasks were presented in white, while the two tasks that could not be chosen were written in gray (cf. **Figure 1A**). There was no time restriction for task indication responses. In case of disallowed task choices in restricted blocks, participants received error feedback (“This task cannot be selected in this trial!”). After a task had been chosen, a second fixation mark was presented for the duration of the IRSI. After that, the imperative stimulus was presented and remained on the screen until the participant’s response or until 2,500 ms had elapsed (cf. **Figure 1B** for an illustration of the single trial procedure). In case of RTs higher than the RT deadline of 2,500 ms, RT feedback (‘too slow’) was presented for additional 1,000 ms; in case of an error, error feedback was presented for additional 1,000 ms.

## RESULTS

The first four blocks (i.e., two restricted and two unrestricted blocks) were considered as practice and therefore excluded from analyzes, as was the first trial of each block. Trials with a task indication time of more than 2,500 ms (1.1%) were discarded, as were trials of the restricted condition in which a task was chosen that was not allowed (0.7%). The same holds for trials following an error (5.3%) or with task execution RTs exceeding 2,500 ms (0.7%). For RT analyzes, error trials were also excluded (3.9%).

### MAIN ANALYZES

Our primary analyzes focused on the comparison of restricted and unrestricted blocks. Regarding restricted blocks, we further distinguished between conditions in which the remaining two candidate tasks included a task repetition (repetition-possible trials) and those in which both candidate tasks implied a task switch (forced-switch trials). The main question to be answered by these analyzes was whether restricting task choice would selectively facilitate task switches, and if so, whether this effect would be consistently observed across all timing conditions. Additionally, we analyzed restricted blocks separately to allow for a direct comparison of our results with those of Demanet and Liefooghe (2014; Experiment 3) who employed restricted blocks only in their experiment.

### TASK EXECUTION RTs IN RESTRICTED AND UNRESTRICTED BLOCKS

Task execution RTs were submitted to an analysis of variance (ANOVA) with the between-participants factor Proportion of Repetition-possible Trials (25 vs. 50%) and the within-participants factors Task Choice (restricted, unrestricted), ERPI/IRSI (100-100; 2,000-100; 100-2,000), and Task Transition (repetition vs. switch). As already mentioned, Task Choice varied between blocks of trials, whereas ERPI/IRSI and Task Transition varied within blocks of trials.

**FIGURE 2:**
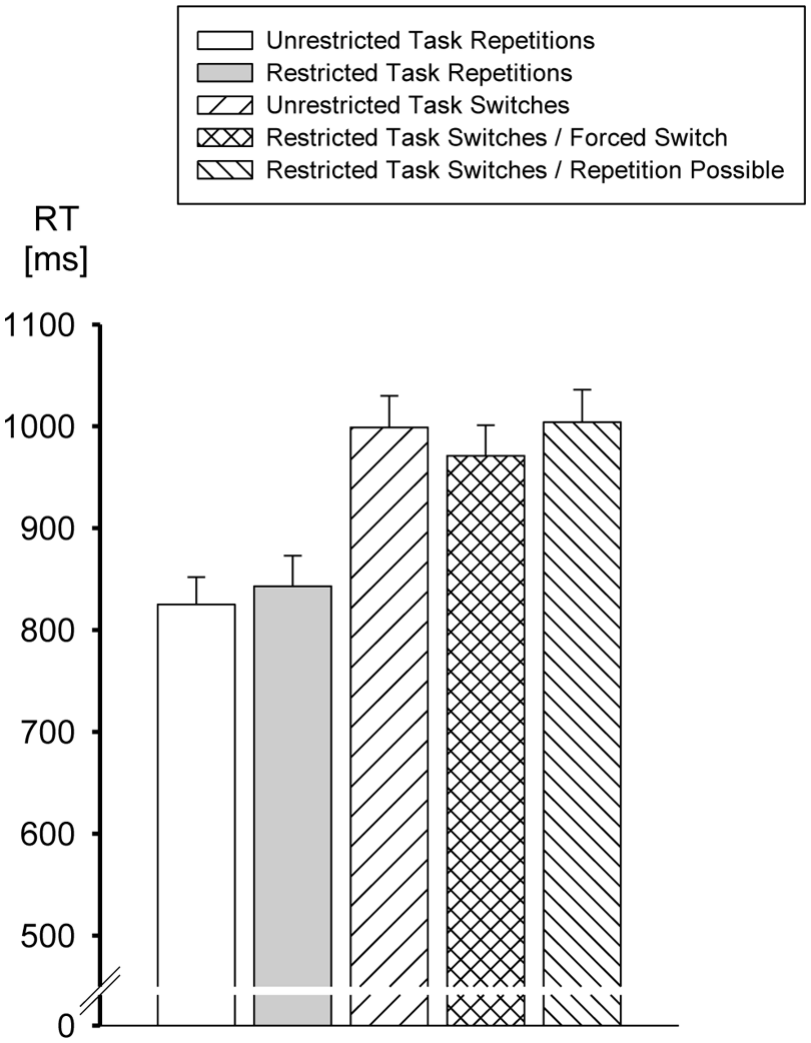
**Mean task execution RTs as a function of Task Transition and Task Choice.** Error bars represent SEM.

Most important, there was a significant interaction of Task Choice and Task Transition, *F*(1,77) = 27.09, MSE = 3,646, *p* < 0.00001. Whereas a restriction of the set of candidate tasks actually increased RTs in task repetition trials from 825 to 843 ms, RTs in switch trials were significantly (Tukey’s *post hoc* test, *p* < 0.001) reduced from 999 to 976 when the set of candidate tasks was restricted from four to two (cf. **Figure 2**). This interaction was not further modulated by either the Proportion of Repetition-possible Trials (*p* > 0.61) or the combination of ERPI/IRSI (*p* > 0.10), nor was there a significant third-order interaction of all factors (*p* > 0.61).

As usual, there was a main effect of Task Transition, *F*(1,77) = 288.40, MSE = 19,340, *p* < 0.00001, indicating a switch cost of 153 ms. Furthermore, there was a significant main effect of ERPI/IRSI, *F*(2,154) = 46.11, MSE 11,938, *p* < 0.00001, that was modulated by Task Choice, *F*(2,154) = 7.38, MSE = 2,520, *p* < 0.001. With unrestricted task choices, mean RT amounted to 860 ms with an ERPI/IRSI combination of 100-100, to 952 ms with a combination of 2,000-100, and to 923 ms with a combination of 100-2,000. With restricted task choices, the corresponding numbers amounted to 873, 948, and 906 ms. Thus, whereas the restriction of task choice increased mean RT with an ERPI/IRSI combination of 100-100, it decreased RT with a combination of 100-2,000. In contrast, mean RT was unaffected by the restriction of task choices with an ERPI/IRSI combination of 2,000-100.

Finally, as indicated by an interaction of Task Transition and ERPI/IRSI, *F*(2,154) = 130.59, MSE = 3,417, *p* < 0.00001, switch costs were affected by the combination of ERPI/IRSI. These amounted to 240 ms with an ERPI/IRSI combination of 100-100, to 106 ms with an ERPI/IRSI combination of 2,000-100, and to 113 ms with a combination of 100-2,000. This was mainly due to particularly fast task repetition RTs with the 100-100 combination (747 ms), which compares to repetition RTs of 897 and 858 ms with combinations 2,000-100 and 100-2,000 (cf. **Table 1**). No other effect approached significance in this analysis (all *p*’s > 0.26).

**Table 1:** Mean task execution Reaction times (RTs) and error rates (ERs) as a function of ERPI-IRSI combination, Task Transition, and Task Choice (SEM in parentheses).

		ERPI-IRSI					
		Task Repetition			Task Switch		
		100-100	100-2,000	2,000-100	100-100	100-2,000	2,000-100
RT	Unrestricted	726 (14)	860 (19)	888 (18)	994 (18)	986 (19)	1016 (19)
	Restricted	767 (16)	855 (17)	905 (17)	980 (18)	956 (18)	991 (19)
ER	Unrestricted	1.7 (0.3)	3.8 (0.4)	2.9 (0.4)	4.7 (0.4)	5.1 (0.5)	4.3 (0.4)
	Restricted	1.7 (0.3)	2.5 (0.4)	2.6 (0.5)	4.5 (0.4)	4.5 (0.4)	4.1 (0.3)

So far, our analysis yielded evidence for a selective decrease of RTs in switch trials when the number of tasks to choose from was restricted from 4 to 2. In order to locate the source of this effect, we subdivided switch trials from restricted blocks into forced switches (i.e., both tasks to choose from implied a task switch) and repetition-possible trials (i.e., the task not chosen was a repetition). We then analyzed task switch RTs as a function of an extended Task Choice factor (unrestricted, restricted – repetition-possible, restricted – forced-switch) and ERPI/IRSI. The main effect of Task Choice reached statistical significance, *F*(2,156) = 10.38, *p* < 0.0001, MSE = 7,160. However, as indicated by Tukey’s *post hoc* test, task switch RTs were significantly smaller only for the forced-switch trials of the restricted condition compared to both switch RTs for the unrestricted condition and for the repetition-possible trials of the restricted condition (*p*’s < 0.001), whereas there was no difference between the latter two conditions (*p* > 0.82). Furthermore, apart from a main effect of ERPI/IRSI, *F*(2,156) = 6.49, *p* < 0.01, MSE = 11,715, Task Choice significantly interacted with ERPI/IRSI, *F*(4,312) = 2.80, *p* < 0.05, MSE = 3,135. Tukey’s *post hoc* tests revealed that this interaction was due to the observation that in the unrestricted condition RTs were higher (*p* < 0.05) with ERPI/IRSI of 2,000-100 ms (1,016 ms) than with an ERPI/IRSI of 100-2,000 (987 ms), with the 100-100 condition falling in between (994 ms). The same holds for forced switches, with RTs of the 2,000-100 condition, amounting to 987 ms, being significantly (*p* < 0.01) higher compared to the 100-2,000 condition (951 ms), with the 100-100 condition lying in between (974 ms). Finally, in repetition-possible trials RTs significantly (*p*’s < 0.001) differed between ERPI/IRSI combinations of 100-100 (1,021 ms) and 100-2,000 (975 ms), and between the latter condition and ERPI/IRSI 2,000-100 (1,014 ms).

The observation that the reduction of task switch RTs in the restricted conditions was exclusively observed in the forced-switch trials but absent in the repetition-possible condition runs counter an interpretation in terms of an establishment of antagonistic constraints. An establishment of antagonistic constraints should be triggered solely by the reduction of the number of candidate tasks from 4 to 2 irrespective of whether the remaining to tasks include a task repetition or not. This pattern was observed by Kleinsorge and Scheil (2013) with cued task switching but obviously failed to materialize with the VTS procedure of the present experiment.

### TASK EXECUTION ERs IN RESTRICTED AND UNRESTRICTED BLOCKS

Task execution ERs were analyzed by the same ANOVA as task execution RTs. This analysis yielded significant main effects of Task Choice, *F*(1,77) = 7.71, MSE = 0.00052, *p* < 0.01, ERPI/IRSI, *F*(2,154) = 6.91, MSE = 0.00085, *p* < 0.01, and Task Transition, *F*(1,77) = 84.45, MSE = 0.00112, *p* < 0.000001. ERs were higher with unrestricted (3.7%) compared to restricted (3.3%) task choices. ERs for the three combinations of ERPI/IRSI amounted to 3.1% (100-100), 3.5% (2,000-100), and 4.0% (100-2,000). Task repetitions were associated with fewer errors (2.5%) than task switches (4.5%). These error switch costs differed among the combinations of ERPI/IRSI, *F*(2,154) = 7.33, MSE = 0.00063, *p* < 0.001, amounting to 2.9, 1.5, and 1.6% with ERPI/IRSI combinations of 100-100, 2,000-100, and 100-2,000, respectively. Finally, a marginally significant (*p* < 0.08) interaction of Task Choice and ERPI/IRSI emerged, *F*(2,154) = 2.69, MSE = 0.00057, being due to the fact that restricted task choices went along with a smaller ER (3.5%) than unrestricted task choices (4.5%) with an ERPI/IRSI combination of 100-2,000, whereas with an ERPI/IRSI combination of 100-100 and 2,000-100, ERs were virtually the same (*p*’s < 0.36) with unrestricted and restricted choices (3.2 vs. 3.1% and 3.6 vs. 3.4%, respectively). No other effect approached statistical significance in this analysis (all *p*’s > 0.38).

As for RTs, task switch ERs were analyzed by an additional ANOVA with the factors ERPI/IRSI and the extended Task Choice factor (unrestricted, restricted – repetition-possible, restricted – forced-switch). This analysis only yielded a marginally significant main effect of Task Choice, *F*(2,156) = 2.49, *p* < 0.09, MSE = 0.000811, due to a tendency toward higher ERs for repetition-possible trials [4.9% compared to forced-switch trials (4.3%)] of the restricted condition, with ERs of the unrestricted condition (4.7%) lying in between.

### TASK INDICATION RTs IN RESTRICTED AND UNRESTRICTED BLOCKS

Task indication RTs were analyzed by the same ANOVA as task execution RTs. This analysis yielded significant main effects of Task Choice, *F*(1,77) = 1,034.23, MSE = 18,241, Task Transition, *F*(1,77) = 66.79, MSE = 35,265, and ERPI/IRSI, *F*(2,154) = 18.92, MSE = 8,446, all *p*’s < 0.00001. Task indication RTs were much higher when task choice was restricted (852 ms) rather than unrestricted (570 ms), corroborating the assumption that the restriction of the set of candidate tasks discouraged premature task choices (cf. Demanet and Liefooghe, 2014). Task switches were accompanied with longer indication latencies (761 ms) than task repetitions (661 ms). Furthermore, conditions with a short ERPI of 100 ms were associated with higher indication RTs (717 and 730 ms for conditions with an IRSI of 100 and 2,000 ms, respectively) than the condition with an ERPI of 2,000 (686 ms). As indicated by significant interactions of Task Choice and ERPI/IRSI, *F*(2,154) = 84.65, MSE = 3,776, *p* < 0.00001, Task Choice and Task Transition, *F*(1,77) = 17.48, MSE = 9,617, *p* < 0.0001, as well as a second-order interaction of Task Choice, ERPI/IRSI, and Task Transition, *F*(2,154) = 9.36, MSE = 2,416, *p* < 0.001, the variations induced by ERPI/IRSI were almost entirely due to conditions with unrestricted task choices and more pronounced with task switches compared to repetitions (cf. **Table 2**). The main difference was always between conditions with a short versus a long ERPI, with the latter being associated with smaller indication RTs than the former. This was corroborated by Tukey’s *post hoc* tests indicating that within any combination of Task Choice and Task Transition, significant differences between ERPI/IRSI combinations only showed up with unrestricted choices and between conditions with short versus long ERPIs.

**Table 2:** Mean task indication RTs as a function of ERPI-IRSI combination, Task Transition, and Task Choice (SEM in parentheses).

	ERPI-IRSI						
	Task Repetition			Task Switch		
	100-100	100-2,000	2,000-100	100-100	100-2,000	2,000-100
Unrestricted	549 (19)	566 (20)	485 (12)	641 (20)	647 (21)	532 (15)
Restricted	776 (18)	797 (22)	795 (19)	902 (21)	911 (22)	934 (21)

In a further step, Task Indication RTs were, like Task Execution RTs and ERs, analyzed by an ANOVA with the factors ERPI/IRSI and the extended Task Choice factor (unrestricted, restricted – repetition-possible, restricted – forced-switch). Both main effects turned out to be significant [*F*(2,156) = 8.23, MSE = 5,848, *p* < 0.001 for ERPI/IRSI; *F*(2,156) = 712.95, MSE = 11,137, *p* < 0.00001, for Task Choice], as did their interaction, *F*(4,312) = 55.99, MSE = 2,791, *p* < 0.00001. With ERPI/IRSI 100-100, tasks were indicated much faster in unrestricted blocks (642 ms) than either forced switches (905 ms) or switches in repetition-possible trials (931 ms). A similar pattern emerged with an ERPI/IRSI of 100-2,000 (648 vs. 912 and 923 ms). With an ERPI/IRSI of 2,000-100, however, unrestricted task choices were indicated even faster (532 ms), with the difference between this and the other ERPI/IRSI combinations being significant (*p*’s < 0.001). However, indication RTs did not differ between this condition and the two other ERPI/IRSI combinations either for forced switches (935 ms) or switches in repetition-possible trials (940 ms).

### TASK CHOICE PROPORTIONS

Additionally, the proportion of chosen task repetitions was analyzed by an ANOVA with the within-subjects factors Task Choice (restricted, unrestricted) and ERPI (100 vs. 2,000 ms) and the between-subjects factor Proportion of Repetition-possible Trials (25 vs. 50 %). (An inclusion of IRSI as a factor in this analysis would have made no sense because the IRSI unfolded only after task choices were made.) As indicated by the main effect of ERPI, *F*(1,77) = 59.60, MSE = 0.00100, *p* < 0.0001, the proportion of task repetitions was higher for short (0.25) compared to long (0.22) ERPIs. Furthermore, the main effect of Task Choice was significant, *F*(1,77) = 102.10, MSE = 0.00731, *p* < 0.0001, due to a higher proportion of task repetitions in the unrestricted (0.28) compared to the restricted (0.18) condition. This effect was modulated by the Proportion of Repetition-possible Trials, *F*(1,77) = 25.26, MSE = 0.00731, *p* < 0.0001. Whereas for the unrestricted condition, both groups did not differ between the proportion of task repetitions (0.29 for the group with 25% of possible task repetitions, 0.27 for the 50% group, Tukey’s *post hoc* test: *p* > 0.38), significantly (*p* < 0.01) more repetitions were chosen if this was possible in 50% of the trials (0.22) compared to 25% of possible repetitions (0.15).

On average, participants chose the magnitude task in 24.3% of all trials, the odd/even task in 26.2%. The font task was chosen in 25.3% of all trials, the color task in 24.2%.

### ADDITIONAL ANALYZES

In order to make the replication part of our data accessible for a direct comparison with the results of Demanet and Liefooghe (2014; Experiment 3), we analyzed the corresponding data the same way as reported by these authors. Because, as in the previous analyzes, the Proportion of Repetition-possible Trials had no effect, we collapsed our data across this factor to increase statistical power. (Note that Demanet and Liefooghe (2014) employed only a proportion of 25% repetition-possible trials.)

### REPETITION-POSSIBLE TRIALS

Reaction times and ERs from repetition-possible trials were analyzed as a function of the factors ERPI/IRSI (100-100; 2,000-100; 100-2,000), and Task Transition (repetition vs. switch). RTs on task switches were longer (1,004 ms) than on task repetitions (841 ms), *F*(1,78) = 193.54, MSE = 16,246, *p* < 0.0001. The main effect of ERPI/IRSI was also significant, *F*(2,156) = 17.90, MSE = 10,147, *p* < 0.0001. As indicated by Tukey’s *post hoc* tests, conditions with an ERPI/IRSI combination of 100-100 were associated with significantly (*p* < 0.0001) smaller RTs (894 ms) than conditions with a combination of 2,000-100 (960 ms) and 100-2,000 (913 ms), with the difference between the latter to conditions failing to reach significance (*p* > 0.2). The interaction of both factors was significant, *F*(2,156) = 57.84, MSE = 4,375, *p* < 0.0001 (cf. **Table 3**).

**Table 3:** Mean task execution RTs and ERs of the restricted/repetition-possible condition as a function of ERPI-IRSI combination and Task Transition (SEM in parentheses).

	ERPI-IRSI						
	Task Repetition			Task Switch		
	100-100	100-2,000	2,000-100	100-100	100-2,000	2,000-100
RT	766 (16)	851 (17)	905 (17)	1021 (20)	975 (21)	1014 (20)
ER	1.6 (0.3)	2.5 (0.4)	2.7 (0.5)	4.4 (0.6)	5.1 (0.6)	5.3 (0.7)

Following Demanet and Liefooghe (2014), we tested the bottom–up component by comparing the effect of Task Transition between the ERPI/IRSI combinations of 100-100 and 2,000-100 by using contrasts based on the error term of the interaction of Task Transition and ERPI/IRSI. This contrast turned out to be highly significant, *F*(1,78) = 3,298.45, *p* < 0.0001. This corresponds to the observation that switch costs declined from 255 ms with an ERPI/IRSI combination of 100-100 to 109 ms with an ERPI/IRSI combination of 2,000-100.

The top–down component was tested by contrasting the switch cost with an ERPI/IRSI combination of 2,000-100 with the switch cost observed with an ERPI/IRSI combination of 100-2,000. Replicating the observations of Demanet and Liefooghe (2014), this contrast failed to reach significance, *F*(1,78) = 0.96, reflecting the fact that the switch cost of 125 ms measured with an ERPI/IRSI combination of 100-2,000 was similar to the one obtained with 2,000-100 ERPI/IRSI.

The ANOVA of ERs from repetition-possible trials yielded only a significant main effect of Task Transition, *F*(1,78) = 60.49, MSE = 0.00139, *p* < 0.0001, reflecting the observation that task switches were associated with more errors (4.9%) than task repetitions (2.3%).

In line with the observations from Demanet and Liefooghe (2014), the ANOVA of Task Indication RTs from repetition-possible trials yielded a significant main effect of Task Transition, *F*(1,78) = 73.35, *p* < 0.0001, MSE = 20,426. Task Indication RTs were shorter for task repetitions (795 ms) compared to task switches (933 ms). The main effect of ERPI as well as the two-way interaction between both factors failed to reach significance (*p* > 0.20 and *p* > 0.69, respectively).

The proportion of task repetitions did not differ between long [0.52, comparison 0.50: *t*(78) = 0.87, *p* > 0.38] and short (0.53, comparison 0.50: *t*(78) = 1.61, *p* > 0.12) ERPIs (*p* > 0.24).

### FORCED-SWITCH TRIALS

Reaction times differed across the three timing conditions, *F*(2,156) = 5.96, MSE = 4,387, *p* < 0.01. Mean RT amounted to 974 ms with an ERPI/IRSI combination of 100-100, to 987 ms with a combination of 2,000-100, and to 951 ms with a combination of 100-2,000. Tukey’s *post hoc* test indicated that only the latter two conditions differed significantly from another (*p* < 0.01). In the corresponding analysis of ERs, the effect of ERPI/IRSI failed to reach significance (*p* > 0.19).

For Task Indication RTs, the main effect of ERPI reached statistical significance, *F*(1,78) = 10.64, *p* < 0.01, MSE = 2,490. RTs were shorter for an ERPI of 100 ms (909 ms) compared to an ERPI of 2,000 ms (935 ms).

## DISCUSSION

In this study, we replicated the finding of Demanet and Liefooghe (2014) of a lack of evidence for top–down preparation in VTS when participants were discouraged from choosing a task and prepare for it in advance of the task indication response. This corroborates the conclusion of Demanet and Liefooghe (2014) that top–down control in VTS is rather fragile and susceptible to procedural variations.

The primary motivation for the present study was based on the supposition that the procedure employed in Experiment 3 of Demanet and Liefooghe (2014) may have induced participants to engage in another kind of top–down processes, namely the establishment of antagonistic constraints among the remaining two candidate tasks. At first glance, we replicated the observation of Kleinsorge and Scheil (2013) that reducing the number of candidate tasks from four to two resulted in a reduction of switch costs also in a VTS procedure. However, upon a closer look it turned out that this effect was confined to forced switch trials, whereas repetition-possible trials were not performed faster than switch trials from unrestricted blocks. This finding clearly deviates from the observations of Kleinsorge and Scheil (2013) because in this study the selective facilitation of task switches by a reduction of the number of candidate tasks was also evident in repetition-possible trials. Therefore, the present study points to a functional divergence of externally presented and internally generated cues that effectuate task selection in cued and VTS, respectively.

Apart from demonstrating quite different effects of a very similar manipulation in cued versus VTS, the present experiment yielded some interesting observations with respect to how participants adapted to a restriction of task choice. Although the RT differences between task switches from unrestricted blocks and forced task switches varied as a function of ERPI/IRSI, it was rather consistent across all timing conditions. This observation suggests that the exclusion of task repetitions resulted in an instantaneous change in participants’ representation of the overall situation, suggesting that the distinction between repetition-possible and forced switch trials was very salient to participants. However, this discrimination probably did not affect the amount of subsequent task-specific preparation: Contrasting conditions with ERPI/IRSI 2,000-100 and 100-2,000, a longer IRSI reduced RT by 36 ms with forced switches but also by 39 ms in repetition-possible trials. Rather, it seems that the detection of a forced switch trial resulted in a general easing of transitioning to a new task. Recently, Herd et al. (2014) proposed a mechanism they termed “clearing on gating” in their neural network model of task switching. This mechanism belongs to the shift-specific component of their model of executive functions. It is assumed to be based on signals sent by the basal ganglia to the prefrontal cortex (PFC) and serves to counteract the “stickiness” of representations maintained in the PFC by effecting a rapid clearance and replacement of its current contents when needed. Importantly, they posited this mechanism to be independent of inhibitory processes subserving goal maintenance. Such a distinction may help to explain that the benefit of forced switch trials over both unrestricted and repetition-possible conditions showed no clear dependence on the duration of the IRSI.

Thus, whereas we observed no evidence for an establishment of antagonistic constraints in VTS when the number of candidate tasks was reduced from four to two, we consider the advantage of forced switch trials as an indication of another top–down control process involved in this variant of VTS. Adopting the notion of ‘clearing on gating’ from Herd et al. (2014), this process seems to be switch-specific but distinct from the preparation for a specific task. Rather, the interpolation of a reduction of the set of candidate tasks probably did not just induce participants to postpone task choice [as was intended by Demanet and Liefooghe (2014)] but resulted in an additional reassessment of choice options in terms of the possibility of a task repetition, with this reassessment affecting the residual activation of the previously executed task. This conclusion is also corroborated by a comparison of task indication RTs in restricted and unrestricted blocks, which shows that task indications were slowed down by about 350–400 ms when the number of tasks was reduced from four to two.

Taken together, our observations point to a functional divergence between externally presented and internally generated task cues regarding the effects of a reduction of the set of candidate tasks. Whereas with externally presented task cues a reduction of the number of tasks to two results in an increased efficiency of task switching that we accounted for by the notion of an establishment of antagonistic constraints (cf. Kleinsorge and Scheil, 2013), reducing the number of tasks to choose from in VTS seems to result in the insertion of an additional discrimination between repetition-possible and forced switch trials. This, in turn, leads to a considerable change in the way participants choose among tasks in VTS. At the very least, this should caution about generalizing from conditions with restricted task choices to VTS in general.

To integrate our findings into a broader perspective, the present study adds to our understanding how processes of moving from one task to the next are shaped by constraints of the general situation as well as the kind of cues that ultimately effect the selection of a certain task. Whereas most studies of task switching are concerned with the local transitions among only two tasks, a growing number of studies (e.g., Kleinsorge et al., 2004; Kleinsorge and Apitzsch, 2012; Kieffaber et al., 2013) suggests that the way we switch among two particular tasks is shaped by all other tasks that may be relevant in a certain situation. Seen from a more applied perspective, being confronted with a relatively large number of tasks is certainly more representative of a working environment than a situation in which only the performance of one of two tasks may be required. Furthermore, this research shows that the relative efficiency of certain cues (externally presented, retrieved from memory, or ‘spontaneously’ generated as in VTS) varies as a function of these situational constraints. For example, the study of Kleinsorge and Apitzsch (2012) demonstrated that switching among two tasks proceeds much more efficiently when triggered by external cues, whereas switching among four tasks is at least as efficient when based on memory rather than being triggered externally. The study of Kleinsorge and Scheil (2013) demonstrated that externally triggered switching among four tasks is aided by an insertion of a short-term reduction of the number of candidate tasks to two, whereas the present study failed to yield corresponding evidence in case of VTS. Further research in this direction should yield a comprehensive picture on how situational constraints and procedural factors combine in ways that promote or hinder flexible switching among different tasks. This, in turn, should help to design multi-tasking environments suitable for reducing the strain inherent in any multi-tasking situation.

## Conflict of Interest Statement

The authors declare that the research was conducted in the absence of any commercial or financial relationships that could be construed as a potential conflict of interest.
